# Optic Nerve Sheath Meningioma Masquerading as Optic Neuritis

**DOI:** 10.1155/2016/5419432

**Published:** 2016-01-19

**Authors:** R. Alroughani, R. Behbehani

**Affiliations:** ^1^Division of Neurology, Department of Medicine, Amiri Hospital, P.O. Box 1661, 13041 Sharq, Kuwait; ^2^Neurology Clinic, Dasman Diabetes Institute, P.O. Box 1180, 15462 Dasman, Kuwait; ^3^Al-Bahar Ophthalmology Center, Ibn Sina Hospital, P.O. Box 25427, 13115 Safat, Kuwait

## Abstract

Optic neuritis is a common presentation of demyelinating disorders such as multiple sclerosis. It typically presents with acute painful monocular vision loss, whereas chronic optic neuropathy can be caused by compressive lesions along the anterior visual pathway, genetic, toxic, or nutritional causes. We report an unusual presentation mimicking optic neuritis, which was subsequently diagnosed as optic nerve sheath meningioma (ONSM). Misinterpretation of white matter lesions on MRI of brain and the failure to image the optic nerves at the time of acute loss of vision led to the misdiagnosis of optic neuritis in this case. A comprehensive accurate history and ordering the appropriate imaging modality remain paramount in diagnosing progressive visual deterioration.

## 1. Introduction

Optic nerve sheath meningioma (ONSM) usually arises from the intraorbital part of the optic nerve sheath and accounts for approximately 2% of all orbital tumor [1]. Typically, it affects middle-aged women with an average age of 41 years [2]. It may cause gradual painless visual loss and is typically present for 1–5 years before clinical presentation [3]. Progressive visual loss, optic nerve atrophy, and presence of optociliary collateral vessels, known as Hoyt-Spencer triad, are a classic sign of an ONSM [4]. The most common visual field defect is peripheral constriction although other field defects such as blind spot enlargement, altitudinal field defects, and central scotomas have been described [1, 2, 5]. In patients with primary ONSM, optic disc abnormalities are nearly always visible at the time of presentation (98%) [1]. Chronic disc swelling occurs when the tumor surrounds or compresses the intraorbital part of the optic nerve [4, 6]. Optic atrophy may be subtle and is a late finding as a patient's optic disc swelling resolves, and optociliary collateral vessels may appear on the surface of the disc [1, 6]. The diagnosis of ONSM is usually made on the basis of clinical and radiographic findings [6]. Meningiomas typically display intense homogenous enhancement with gadolinium-enhanced fat-suppression T1-weighted pulse sequences in MRI [7]. We report an unusual presentation mimicking optic neuritis, which was subsequently diagnosed as optic nerve sheath meningioma (ONSM). The aim of this report is to raise the awareness and highlight the diagnostic approach in patients presenting with atypical features.

## 2. Case Presentation

**Figure 1:**
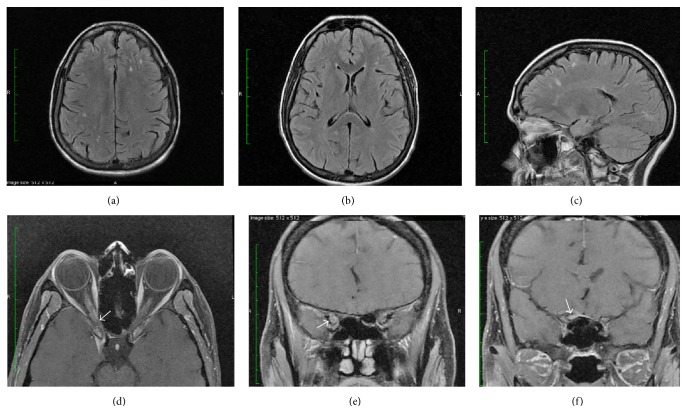
Brain MRI T2 FLAIR axial (a, b) and sagittal (c) scans made at the time of presentation showing multiple nonenhancing hyperintense lesions in the periventricular, deep, and subcortical white matter. MR contrast-enhanced axial (d) and coronal (e, f) sequences of the orbit. The arrows pointed to a small extra-axial lesion with peripheral enhancement extending into the optic canal and apex of orbits surrounding the optic nerve with a small amount of fat stranding this region.

A 47-year-old woman presented with history of acute decrease in vision of the right eye 4 years ago. She has reported that the onset was “sudden” yet with rapid progression within 1 week and was associated with “pain with eye movement.” She denied any previous history of weakness, paresthesia, or bulbar or bowl/bladder symptoms. Review of her records showed that initial neuro-ophthalmological examination four years ago revealed that her visual acuity was hand motion recognition in the right eye and 20/20 in the left eye. She had a large right relative pupillary afferent defect (RAPD). Anterior segment examination was within normal limits and her ocular motility was full with no ocular misalignment or limitation of eye movements. Fundoscopic examination showed normal optic discs and maculae in both eyes. Humphrey visual fields 30-2 showed a large right central scotoma in the right eye and was normal in the left eye. Neurological examination was unremarkable. The initial MRI of the brain reportedly showed multiple nonenhancing T2/FLAIR hyperintense lesions within the periventricular, deep, and subcortical white matter in a nonspecific distribution. Orbital cuts were not performed in the original MRI. The white matter lesions were seen in the subsequent MRI obtained when the patient presented to us (Figures 1(a)–1(c)). There was no infratentorial or spinal lesions. A provisional diagnosis of retrobulbar optic neuritis was made at that time and she had received intravenous methylprednisolone (IVMP) one gram once daily for 5 days without any improvement. She did report that her visual acuity in her right eye had progressed within few weeks to light perception only. Plasmapheresis was instituted 3 weeks after the initial IVMP course with no improvement in her visual acuity. Vasculitic screen including CRP, double stranded DNA, ANA, ENA, and serum angiotensin-converting enzyme along with vitamin B12 levels were within normal limits. Serum anti-neuromyelitis optica antibody was negative. CSF analysis revealed normal cell counts, protein, Gram stain, and absence of oligoclonal bands. A genetic testing for Leber Hereditary Optic Neuropathy (LOHN) was negative. She had a tentative diagnosis of multiple sclerosis by her treating neurologist and was offered disease-modifying therapy but she elected not to start treatment. Three years after her initial presentation, she reported slight progression in her vision without any associated symptoms. When she presented to us her visual acuity was light perception in the right eye and 20/20 in the left eye. We obtained an MRI of the brain and orbit with contrast enhancement, which showed a small extra-axial lesion along the right anterior clinoid process showing peripheral enhancement (Figures 1(d)–1(f)). Enhancing tissue was noted extending into the optic canal and apex of orbits surrounding the optic nerve sheath with a small amount of fat stranding this region. Additionally, there was mild hyperostosis of the planum sphenoidale on the right and a dural tail was noted. A diagnosis of optic nerve sheath meningioma was made. She received fractionated stereotactic radiotherapy and she reported subjective improvement in her vision although her visual acuity in the right eye remains light perception only. Postradiation MRI orbit 6 months later showed slight regression of the extra axial lesion but with persistent enhancement. There is also decreased extension into the right optic canal.

## 3. Discussion

Our case highlighted the difficulty in diagnosing ONSM when presented with acute visual loss in association with painful eye movements mimicking optic neuritis. The unusual presentation can be attributed to the “sudden awareness” rather than the sudden onset in a patient who was not sufficiently cognisant to give an accurate history. The normal fundoscopic examination was also supportive of acute retrobulbar optic neuritis. It was obvious that the MRI findings of white matter lesion had a significant impact on directing the provisional diagnosis toward a demyelinating process, specifically multiple sclerosis. Misinterpretation of the MRI resulted in a delay of the diagnosis and possibly missing the therapeutic window in this patient. In addition, the omission of high-spatial-resolution contrast-enhanced MRI of the orbit with thin sections (less than 3 mm) particularly with progressive loss of vision was also an important factor in the delay of diagnosis. Progressive visual loss and unresponsiveness to high-dose steroids are usual red flags in optic neuritis secondary to multiple sclerosis and they usually point toward more serious diagnoses such as neuromyelitis optica or orbital pathology. Although OSNM typically presents with slowly progressive visual loss, atypical acute presentation leading to an initial diagnosis of “optic neuritis” has been reported [8–10]. Jackson et al. reported six cases of intracanalicular optic nerve sheath meningioma in which the diagnosis was missed for more than 1 year after clinical presentation. Clinical features led to a misdiagnosis of optic neuritis in all cases. The use of inappropriate imaging protocols in these cases led to the delay of the diagnosis, which was eventually made on the basis of high-spatial-resolution contrast-enhanced MR findings. Occasionally, it might be difficult to determine where the meningioma arose since sphenoidal area is another potential location and very close to the optic canal. However, bony changes are more prominent with sphenoidal than optic nerve sheath lesions. The authors recommended the use of fat-suppression sequences in combination with contrast enhancement whenever possible [10].

In summary, ONSM may present with atypical features such as acute painful visual loss in association with normal fundoscopic examination. Lack of recovery or progressive loss of vision should prompt the clinician to explore alternative diagnoses other than typical demyelinating optic neuritis. Finally selecting the appropriate imaging protocol in the setting of progressive loss of vision, particularly high resolution contrast MRI of the orbit with fat-suppression sequences, can be crucial in making the correct diagnosis.
